# FOXO3/Rab7-Mediated Lipophagy and Its Role in Zn-Induced Lipid Metabolism in Yellow Catfish (*Pelteobagrus fulvidraco*)

**DOI:** 10.3390/genes15030334

**Published:** 2024-03-04

**Authors:** Fei Xiao, Chuan Chen, Wuxiao Zhang, Jiawei Wang, Kun Wu

**Affiliations:** 1 College of Marine Sciences, South China Agricultural University, Guangzhou 510642, China; xiaofei@stu.scau.edu.cn (F.X.); cc18190392889@163.com (C.C.); 18143604051@163.com (J.W.); 2 Nansha-South China Agricultural University Fishery Research Institute, Guangzhou 510642, China; 3 College of Marine and Biology Engineering, Yancheng Institute of Technology, Yancheng 224051, China; zhangwx257258@163.com

**Keywords:** *rab7*, FOXO3, promoter analysis, lipophagy, lipid metabolism, Zn

## Abstract

Lipophagy is a selective autophagy that regulates lipid metabolism and reduces hepatic lipid deposition. However, the underlying mechanism has not been understood in fish. In this study, we used micronutrient zinc (Zn) as a regulator of autophagy and lipid metabolism and found that Ras-related protein 7 (*rab7*) was involved in Zn-induced lipophagy in hepatocytes of yellow catfish *Pelteobagrus pelteobagrus*. We then characterized the *rab7* promoter and identified binding sites for a series of transcription factors, including Forkhead box O3 (FOXO3). Site mutation experiments showed that the −1358/−1369 bp FOXO3 binding site was responsible for Zn-induced transcriptional activation of *rab7*. Further studies showed that inhibition of *rab7* significantly inhibited Zn-induced lipid degradation by lipophagy. Moreover, *rab7* inhibitor also mitigated the Zn-induced increase of *cpt1α* and *acadm* expression. Our results suggested that Zn exerts its lipid-lowering effect partly through *rab7*-mediated lipophagy and FA β-oxidation in hepatocytes. Overall, our findings provide novel insights into the FOXO3/*rab7* axis in lipophagy regulation and enhance the understanding of lipid metabolism by micronutrient Zn, which may help to reduce excessive lipid accumulation in fish.

## 1. Introduction

Autophagy is a dynamic and highly inducible degradative system that eliminates damaged or dysfunctional cytosolic components, recycles cellular nutrients, and upholds intracellular homeostasis [1]. Autophagy also has a significant role in the regulation of lipid metabolism [2]. Lipid droplets (LDs) undergo degradation via a specialized autophagy process known as lipophagy. This process involves the encapsulation of LDs by autophagosomes, their fusion with lysosomes to form autolysosomes, and subsequent hydrolysis into glycerol and fatty acids by lysosomal acid lipases [3,4]. Inhibiting lipophagy leads to increased lipid accumulation and reduced lipolysis in hepatocytes [2,5]. Conversely, moderate lipophagy supplies free fatty acids (FFAs) for mitochondrial β-oxidation, fosters development, sustains cellular energy balance and averts lipotoxicity [6,7,8]. However, overactive lipophagy can cause the excessive breakdown of necessary cellular components, resulting in cell damage or even cell death [9]. Therefore, lipophagy needs to be maintained at an appropriate level, as defective lipophagy can precipitate various metabolic disorders such as obesity, diabetes, atherosclerosis, and fatty liver [4].

The molecular cascade regulating lipophagy is complex, involving numerous proteins and pathways that are not yet fully understood. Our prior research posits that *rab7*, a small GTPase of the Rab family, plays an indispensable role in lipophagy, particularly under a high-fat diet [5]. Upon activation, *rab7* facilitates the docking and fusion of autophagosomes and lysosomes, enabling lipid delivery to lysosomes for degradation [10]. Additionally, *rab7* coordinates with other proteins to manage lysosomal biogenesis and the positioning of autophagosomes and lysosomes [11]. In eukaryotes, promoters contain multiple cis-acting elements that transcription factors can bind to, initiating transcription and thus regulating gene expression. Mammalian cell studies have identified FOXO3, a member of the Forkhead box O (FOXO) family, as a key upstream regulator of *rab7*, targeting the *rab7* promoter [12,13]. In teleosts, our recent findings also suggest that the FOXO3 signal may be a vital link between dietary nutrition and lipophagy [8]. Nonetheless, the paucity of genetic data on *rab7* hampers further investigation into the molecular regulatory mechanisms of lipophagy.

The yellow catfish (*P. fulvidraco*), extensively farmed in China and other Asian countries for its high market value, often develops severe fatty liver syndrome under intensive farming conditions. This condition, detrimentally impacting its health, is primarily attributed to lipophagy disorder and subsequent lipid accumulation [5,14]. Consequently, investigating the molecular underpinnings of lipophagy and strategies to mitigate lipid deposition is of paramount importance. Zinc (Zn), a vital micronutrient, plays a significant role in numerous biochemical processes in vertebrates, including fish. A burgeoning number of studies underscore Zn’s influence on lipid metabolism [15,16,17,18], with recent research highlighting its regulatory effect on lipophagy and lipolysis, thereby impacting lipid accumulation [8,14]. Given the critical function of *rab7* in lipophagy, we postulate that it is a mediator in the Zn-induced lipophagy process and helps to prevent lipid accumulation. In this study, we delineated the *rab7* promoter region in *Pelteobagrus fulvidraco* with its interaction with FOXO3 and the effects of the lipophagy pathway in response to Zn signaling. Our findings provide novel insights into *rab7*’s role in lipophagy regulation, contributing to the understanding of lipid metabolism control and offering theoretical guidance for reducing excessive lipid accumulation in vertebrates.

## 2. Materials and Methods

### 2.1. Experimental Animals and Reagents

Healthy yellow catfish (average weight of about 50 g) were acquired from a local commercial aquaculture facility in Guangzhou, China. We anesthetized yellow catfish using MS-222 (80 mg/L; Sigma-Aldrich, St. Louis, MO, USA). According to our study, hepatocytes were isolated from healthy yellow catfish [14]. HepG2 cell lines originated from our college’s Cell Resource Center. Both Dulbecco’s Modified Eagle Medium (DMEM) and fetal bovine serum (FBS) were procured from Pricella (Procell Life Science & Technology Co., Ltd., Wuhan, China). 

### 2.2. Promoter Cloning and Plasmid Construction

We employed the high-efficiency thermal asymmetric interlaced PCR method [19] to clone promoter sequences and followed the protocols developed in previous research [20,21]. For primer details, consult Supplementary Table S1. Luciferase reporter constructs were generated from purified PCR products and pGl3-Basic vectors (Qiyunbio, Nanchang, China), and the products were ligated using the ClonExpress II One-Step Cloning Kit (Transgen, Beijing, China). The plasmids, named based on their proximity to the TSS (transcription start sites), include pGl3−1600/+67 of the *rab7* vector. Utilizing the pGl3−1600/+67 vector as a template, we created pGl3−382/+67, pGl3−795/+67, and pGl3−1206/+67 vectors using the Erase-a-Base system (Promega, Madison, WI, USA). Supplementary Table S2 shows the primer sequences used to construct the plasmids.

### 2.3. Sequence Analysis and Activities Assays of Luciferase

MatInspector (http://www.genomatix.de (accessed on 10 October 2023)) and JASPAR (http://jaspar.genereg.net (accessed on 10 October 2023)) were used for predictive analysis of transcription factor binding sites (TFBS). Supplementary Table S3 shows the reference sequences for these binding sites. Activity assays and plasmid transfections align with protocols delineated in recent publications [20,21]. In brief, HepG2 cells were incubated in DMEM (10% FBS) (Beyotime Biotechnology, Shanghai, China) within a 5% CO_2_ atmosphere at 37 °C. For transient transfection, cells were cultured with a density of 1.2 × 10^5^ in 24-well plates for 24 h to achieve 70–80% confluence. Transfections were performed by PEI Transfection Reagent (Transgen, Beijing, China), and the reporter plasmid was co-transfected with 35 ng of pRL-TK as a control. Four hours post-transfection, the medium was replaced with either DMEM (10% FBS) or DMEM supplemented with 60 μM Zn. After a 24-h incubation, relative luciferase activity was quantified using the Dual-Luciferase Reporter Assay System, adhering to the manufacturer’s instructions.

### 2.4. Hepatocyte Culture and Treatments

The hepatocyte experiment of *Pelteobagrus fulvidraco* consists of two parts. In the first part, hepatocytes were incubated in medium with three different concentrations of control (without additional ZnSO_4_), L-Zn (20 μM ZnSO_4_), and H-Zn (60 μM ZnSO_4_) to explore the activation of Zn on *rab7* and lipophagy. Based on the results of the first part, the experimental design in the second part is as follows: control (without additional ZnSO_4_), H-Zn (60 μM ZnSO_4_), CID (10 μM CID 1067700, a specific *rab7* GTPase inhibitor), Zn + CID (60 μM ZnSO_4_, 10 μM CID 1067700), to explore the regulatory effects of *rab7* on lipophagy and lipid metabolism. The inhibitor concentrations were chosen based on our preliminary trials and corroborating literature [22,23]. Yellow catfish hepatocytes were isolated as previously detailed [5], with each condition replicated thrice. After 48 h, the cells were harvested for subsequent analysis.

### 2.5. Site Mutation Assays of FOXO3 Binding Sites on the rab7 Promoter

To pinpoint the FOXO3 binding sites in the rab7 promoter, we conducted site mutation assays. Using the pGl3−1600/+67 vector as a template, we performed site-directed mutagenesis with the QuickChange II Site-Directed Mutagenesis Kit (Vazyme Biotech Co., Ltd., Nanjing, China). Supplementary Table S4 shows the primers for mutagenesis. The resulting constructs, Mut-FOXO3-1, Mut-FOXO3-2, and Mut-FOXO3-3, were co-transfected with pRL-TK into HepG2 cells, following the methods outlined above. Based on the results from *Pelteobagrus fulvidraco* primary hepatocytes, we incubated the HepG2 cells in the treatment group with 60 μM ZnSO_4_ and the cells in the control group with normal medium. We harvested the cells after 24 h of incubation and measured luciferase activity.

### 2.6. Zn, LD, Autophagic Vesicles, and Triglycerides Content in Yellow Catfish Hepatocytes 

We measured intracellular Zn^2+^ concentration by incubating cells with Newport Green DCF (Beyotime Biotechnology, Shanghai, China) for 20 min. We performed intracellular LD staining by incubating cells with Bodipy 493/503 (Beyotime Biotechnology, Shanghai, China) for 20 min. We detected autophagic vesicles by incubating cells with either acridine orange (Beyotime Biotechnology, Shanghai, China) or LysoTracker Red (Beyotime Biotechnology, Shanghai, China) for 60 min. We imaged fluorescence using a laser scanning confocal microscope (Zeiss, Germany) and quantified fluorescence intensity using flow cytometry (Beckman, Brea, CA, USA). We analyzed intracellular triglycerides (TG) content using commercial assay kits (Biosharp Biotechnology, Hefei, China). 

### 2.7. Quantitative Real-Time PCR (qPCR) Assay

Extraction of total RNA was performed with Trizol reagent (Transgen, Beijing, China). Using 1% denaturing agarose gel electrophoresis and a NanoDrop 2000 spectrophotometer (Thermo Fisher Scientific, Waltham, MA, USA), the quality and concentration of total RNA were measured, respectively. A reverse transcription kit (Transgen, Beijing, China) was used to synthesize cDNA from total RNA. Real-Time Quantitative PCR (Q-PCR) assays were detected by Sybr Green (MilliporeSigma, USA) and performed with the CFX96 Real-Time PCR system (Bio-rad, Hercules, CA, USA). Based on geNorm software [24], we selected the two most stable genes (*β-actin* and *elfa*) from eight housekeeping genes (*hprt*, *tuba*, *elfa*, *rpl7*, *tbp*, *b2m*, *gapdh*, and *β-actin*) under the experimental conditions. Supplementary Table S5 shows the primers. The 2^−ΔΔCT^ method was used to calculate the relative expression of genes [25].

### 2.8. Statistical Analysis

Results are presented as mean ± S.E.M (standard errors of means). Before analysis, the normality of the distribution was assessed using the Kolmogorov–Smirnov test, and the homogeneity of the variance was assessed using the Bartlett test. One-way ANOVA (Duncan’s multiple range test) or Student’s *t*-test was performed using SPSS 27.0 software (SPSS, Chicago, IL, USA). Significance was defined as *p* < 0.05.

## 3. Results

### 3.1. Zn Reduces TG Content in Hepatocytes by Inducing Lipophagy

**Figure 1:**
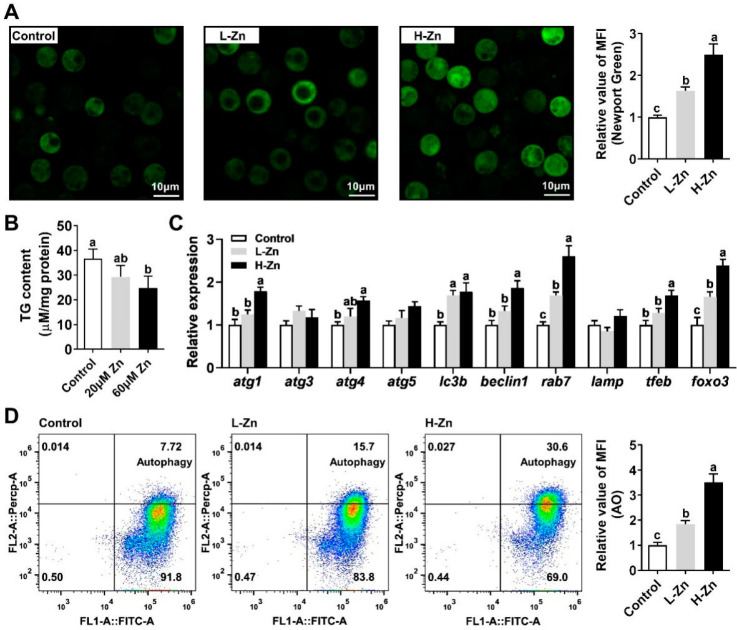
Zn reduces TG content in hepatocytes by inducing lipophagy. (**A**) Intracellular Zn concentration. (**B**) TG Content. (**C**) mRNA levels involving autophagy. (**D**) Relative mean fluorescence intensity of AO (acridine orange). Outcomes are given as mean ± S.E.M (n = 3). Distinct lowercase letters above the bars denote remarkable differences (*p* < 0.05).

Our initial investigation focused on the response of rab7 and autophagy to Zn signaling. We observed that escalating Zn incubation levels led to a significant rise in intracellular Zn concentration within hepatocytes (Figure 1A). Notably, compared with the control, 60 μM Zn treatment substantially reduced TG content. (Figure 1B). Further analysis showed that Zn markedly upregulated the mRNA expression of autophagy-related genes tfeb, atg4, beclin1, lc3b, and atg1, while it did not affect atg3, atg5, and lamp. Crucially, the mRNA expression of rab7 and foxo3 escalated in tandem with Zn concentration (Figure 1C). Additionally, autophagy was detected through flow cytometric analysis, which demonstrated that Zn treatment significantly enhanced the red–green fluorescence ratio, indicative of autophagy enhancement (Figure 1D). Collectively, these results suggest that Zn reduces TG content in hepatocytes by inducing lipophagy, with rab7 potentially playing a pivotal role.

### 3.2. The rab7 Promoter Possesses Multiple Potential FOXO3 Binding Sites

**Figure 2:**
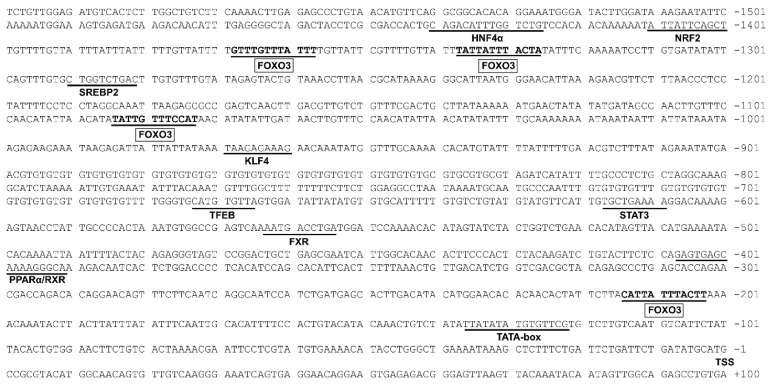
Nucleotide sequence of yellow catfish Rab7 promoter. Numbers are relative to the transcription start site (+1). Underlining indicates putative transcription factor binding sites. The highlighted sequences are potential transcription factor binding sites for FOXO3. The first nucleotide of 5′ cDNA of Rab7 was designated as +1. TSS: transcription start site.

Considering the pivotal role of rab7 in lipophagy, we isolated its promoter for in-depth analysis. This study successfully cloned the 1600 bp rab7 promoter from yellow catfish (Figure 2). We discovered multiple potential FOXO3 binding sites on the rab7 promoter. The core promoter element TATA-box (TBP) was located at positions −123 bp to −137 bp. Furthermore, we projected the binding sites of various transcription factors at the rab7 promoter, including HNF4α (Hepatocyte nuclear factor 4α), FXR (Farnesoid X receptor), NRF2 (Nuclear factor erythroid2-related factor 2), SREBP2 (Sterol-regulatory element-binding protein 2), TFEB (transcription factor EB), KLF4 (Kruppel-like factor 4), PPARα/RXR (peroxisome proliferator-activated receptor α/retinoid X receptor), and STAT3 (signal transducer and activator of transcription 3).

### 3.3. The rab7 Promoter Depends on Specific Regions to Respond to Zn Signals

**Figure 3:**
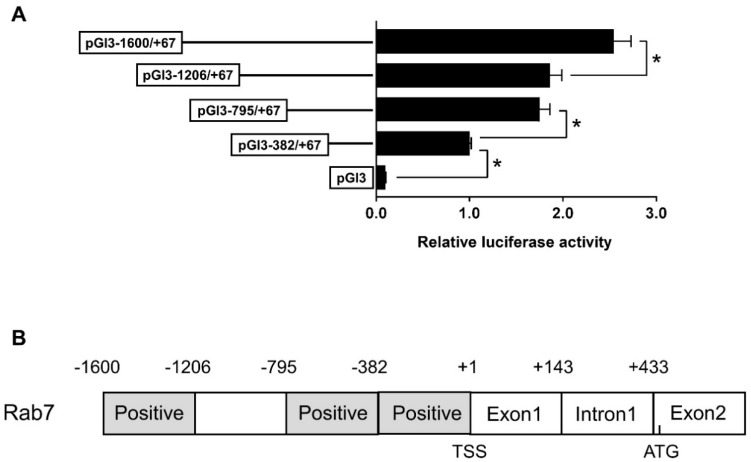
The 5′ unidirectional deletion experiments of the Rab7 promoter region of yellow catfish. (**A**) Values imply the ratio of activities of firefly to Renilla luciferase and were normalized to the control plasmid. Outcomes are given as mean ± S.E.M (n = 3). Asterisk (*) means marked variation between the two groups (*p* < 0.05). (**B**) The schematic diagram of Rab7 gene structure. The first nucleotide of 5′ cDNA of Rab7 was designated as +1. TSS: transcription start site. Positive: the region that positively regulated the promoter activity. ATG: translation initiation site.

We further performed the 5′-deletion assay of the rab7 promoter. We synthesized four plasmids with different size fragments for the assay. Sequence deletions from −1600 bp to −1206 bp and from −795 bp to −382 bp markedly decreased luciferase activity, whereas deletion from −1206 bp to −795 bp did not markedly affect luciferase activity (Figure 3A). These findings suggest the presence of transcriptional binding sites within the −795 bp to −382 bp and −1600 bp to −1206 bp regions that positively regulate promoter activity (Figure 3B).

**Figure 4:**
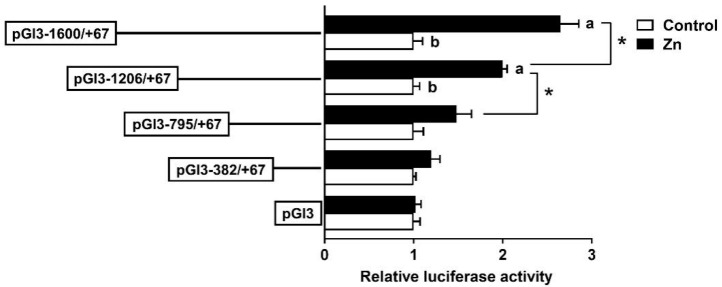
After 60 μM Zn treatment, the 5′ unidirectional deletion assays for promoter regions of *rab7*. Values imply the ratio of activities of firefly to Renilla luciferase and were normalized to the control plasmid. Outcomes are given as mean ± S.E.M (n = 3). Asterisk (*) indicates remarkable differences between different 5′ unidirectional deletion plasmids under the same treatment (*p* < 0.05). Different letters indicate remarkable differences between the different treatments in the same plasmid (*p* < 0.05).

To examine the promoter response to Zn, HepG2 cells were incubated with 60 μM Zn^2+^ for 48 h, followed by a 5′-deletion assay. Zinc markedly increased the luciferase activities of pGl3−1600/+67 and pGl3−1206/+67 but had no effect on pGl3−795/+67 and pGl3−382/+67 compared to the control. In the Zn-treated group, the sequence deletion between −1600 bp and −795 bp of the rab7 promoter showed remarkable influences on luciferase activity. Yet, further deletion to −382 bp did not show significant effects (Figure 4). The result indicated that the rab7 promoter may depend on specific regions from −1600 bp to −1206 bp to respond to Zn signals.

### 3.4. The FOXO3 Binding Site Regulated rab7 Promoter Activity

**Figure 5:**
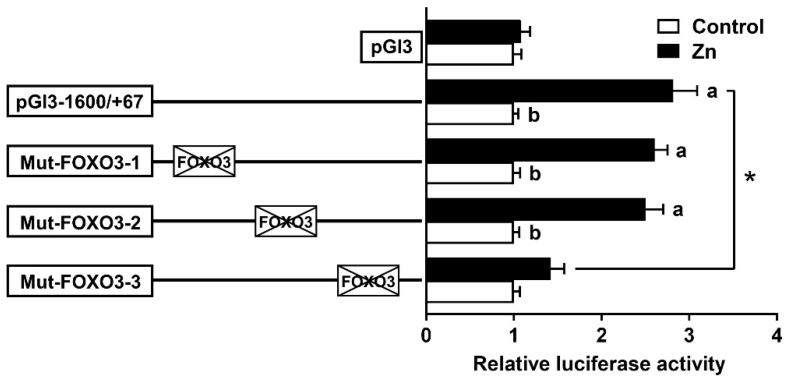
After site-directed mutagenesis, assays of predicted FOXO3 binding sites. Values imply the ratio of activities of firefly to Renilla luciferase and were normalized to the control plasmid. Outcomes are given as mean ± S.E.M (n = 3). Asterisk (*) means that there are remarkable differences between different mutant plasmids under the same treatment conditions (*p* < 0.05). Different letters indicate remarkable differences between the different treatments in the same plasmid (*p* < 0.05).

According to the results of the 5′ deletion assay, we conducted site mutation analysis by using the pGl3−1600/+67 plasmid. The mutation of the −1074/−1085 and −1327/−1338 FOXO3 binding sites (Mut-FOXO3-1 and Mut-FOXO3-2) did not affect the Zn-induced elevation of luciferase activity, showing that these sites were not involved in the rab7 transcriptional response to Zn. Conversely, the mutation of the −1358/−1369 FOXO3 binding site (Mut-FOXO3-3) significantly reduced the Zn-induced luciferase activity (Figure 5). The −1358/−1369 bp FOXO3 site probably has a crucial role in mediating the Zn-induced upregulation of rab7 promoter activity.

### 3.5. rab7 Mediated Zn-Induced Lipophagy to Reduce Lipid Accumulation

**Figure 6:**
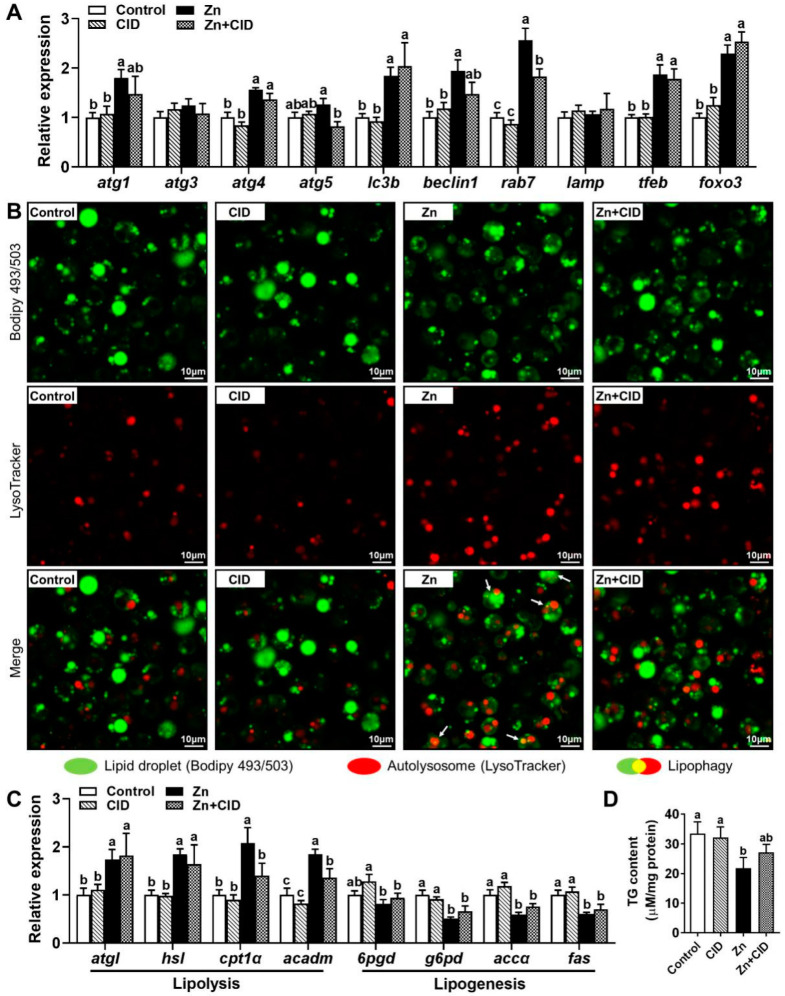
Effects of CID 1067700 on Zn-induced lipophagy. (**A**) mRNA levels involved in autophagy. (**B**) The co-localization analysis of the autolysosomes and the lipid droplets in hepatocytes co-stained with LysoTracker and BODIPY 493/503. (**C**) mRNA levels involving lipid metabolism. (**D**) TG Content. Outcomes are given as mean ± S.E.M (n = 3). Distinct lowercase letters above the bars indicate remarkable differences (*p* < 0.05).

We used the rab7 inhibitor CID 1067700 to elucidate the regulatory mechanism of rab7 on lipophagy and lipid metabolism. We performed a co-localization analysis of the autolysosomes and the lipid droplets (LDs) in hepatocytes co-stained with bodipy 493/503 (green) and LysoTracker (red). The analysis showed that 60 µM Zn treatment increased lipophagy (yellow), whereas Zn + CID co-treatment decreased it (Figure 6B). Zn treatment also significantly upregulated the mRNA levels of lipophagy-related genes (atg1, atg4, atg5, lc3b, beclin1, rab7, tfeb, and foxo3) compared to Zn + CID co-treatment (Figure 6A). Genes implicated in lipid metabolism, Zn upregulated the lipolysis genes (acadm, hsl, cpt1α,batgl) and downregulated the lipolysis genes (fas, g6pd, accα) (Figure 6C). And the increased expression of cpt1α and acadm induced by Zn was suppressed by rab7 inhibitor. In addition, Zn treatment significantly reduced TG content, but Zn + CID co-treatment reversed this effect (Figure 6D). These results suggest that rab7 mediated Zn-induced lipophagy to reduce lipid accumulation in hepatocytes of *Pelteobagrus fulvidraco*.

## 4. Discussion

As vital nutrients, lipids play an essential role in metabolic processes. The inability to preserve lipid homeostasis in fish increases the risk of fatty liver disease, leads to compromised lipid profiles, and hinders various physiological processes [5,26]. Numerous studies have demonstrated that lipophagy, a specialized form of autophagy, can modulate lipid metabolism and decrease lipid accumulation [4,27,28,29]. Previous studies indicated that *rab7* and *foxo3* are important regulators of lipophagy-related genes in *Pelteobagrus fulvidraco* [5,8], but direct evidence has not been explored. Given that Zn is an autophagy activator in fish [14,30], we focused on the structure and function of the *rab7* promoter and investigated the molecular regulatory mechanism of *rab7* in Zn-induced lipophagy in *Pelteobagrus fulvidraco*. 

We first investigated the effect of Zn on lipophagy in yellow catfish. Acridine orange staining and gene expression results indicated that Zn, especially at relatively high concentrations, activated autophagy, which is consistent with previous studies [14,30,31]. *rab7* is a key component of lysosomes and late endosomes and mediates LD and lysosomal fusion, which is essential for lipophagy [32,33]. In this study, *rab7* expression correlated positively with intracellular Zn concentration, implying that autophagy may participate in lipid degradation. This was also supported by the reduced cellular TG content in the treatment groups (Figure 1B). Notably, the expression changes of foxo3 in different treatment groups matched those of *rab7* (Figure 1C). Studies in mammals have revealed that FOXO3 is a transcription factor for several autophagy genes, including *rab7* [12,34,35,36]. Thus, the Foxo3-*rab7* pathway may have a significant role in the lipophagy process of yellow catfish. However, more direct evidence is needed.

An initial step in exploring the transcription initiation mechanism begins with the identification of the core promoter, situated at the closest end of the start codon and containing the RNA polymerase binding site [37]. In the present study, the structure and function of the rab7 promoter of *Pelteobagrus fulvidraco* were cloned and characterized for the first time. In mammals, the CAAT-box and TATA-box were usually located upstream near the TSS and facilitated the docking of the RNA polymerase transcription complex [38]. In this study, one classic TATA box binding site was identified in the core *rab7* promoter region (Figure 2). The identification of transcription factor binding sites is useful in deciphering the regulatory mechanisms of genes. We found that the luciferase activity of the rab7 promoter did not change while the sequence was extended from −1206 bp to −795 bp, possibly due to the absence of a critical binding site in the region. In contrast, deleting the sequence from −1600 bp to −1206 bp or from −795 bp to −382 bp significantly reduced the luciferase activity. Further analysis revealed that this region contained a cluster of TFBSs, such as FOXO3, STAT3, TFEB, PPARα, and FXR (Figure 2). Reportedly, these transcription factors regulate the expression of *rab7* in mammals [36,39,40,41,42]. Consequently, the putative transcription factors in these two regions are likely positive regulators of the *rab7* gene (Figure 3B). As expected, we found TFEB on the *rab7* promoter. TFEB is a master regulator of many genes in the autophagy–lysosomal pathway [43,44], which is consistent with the important role of *rab7* in autophagy. In addition, many of these predicted transcription factors are involved in lipid metabolism, such as PPARα/RXR, FXR HNF4α, and SREBP2, showing that *rab7* may play a vital role in the regulation of lipid homeostasis [33,45]. NRF2 and STAT3 are key regulators of cellular antioxidant and immune responses. Our results suggested that *rab7* may be essential for maintaining the antioxidant and immune balance, as supported by other studies [46,47,48,49,50]. Most importantly, we predicted multiple FOXO3 transcription factor binding sites in the *rab7* promoter region (Figure 2), confirming our initial hypothesis. In summary, the *rab7* promoter has many binding sites that are involved in various cellular functions. *rab7* may have more potential functions than we anticipated, and further exploration is warranted.

We investigated next whether FOXO3 plays a regulatory role via these response elements in response to zinc signaling. Zn incubation significantly increased *rab7* promoter activity from −1600 bp to −795 bp, indicating that Zn promotes *rab7* expression (Figure 4), in agreement with a previous study [31]. Recently, studies have emphasized the significance of the FOXO3 pathway in the regulation of genes involved in lipophagy [12,13,51], especially in the presence of zinc [8]. Given that the three putative FOXO3 binding sites were located between −1600 bp and −795 bp, this promoter sequence might represent a key region in responding to Zn signaling and regulating lipophagy in *Pelteobagrus fulvidraco*. In this study, mutations at the −1358/−1369 FOXO3-binding site (Mut-FOXO3-3) but not at the −1074/−1085 or −1327/−1338 FOXO3-binding sites (Mut-FOXO3-1 and Mut-FOXO3-2), decreased the Zn-induced rise in promoter activity (Figure 5). Information on the interaction between FOXO3 and *rab7* in fish is extremely scarce. Our results implied that the −1358/−1369 bp sequence mediated *rab7* promoter activity and that Zn incubation facilitated the binding of FOXO3 to this site. In mammals, Niu et al. [13] showed that the target gene of FOXO3 was *rab7* and supplied the target promoter sequence. This concurs with our results. Thus, the −1358/−1369 bp FOXO3 site probably plays a vital function in the upregulation of *rab7* expression in response to Zn-induced upregulation.

After determining that FOXO3 stimulates *rab7* transcription by binding to its promoter, we next exploited their effects in the Zn-induced lipophagy and regulation of lipid metabolism with *rab7* inhibitor (CID). Compared to single Zn treatment, Zn + CID co-treatment significantly downregulated the mRNA level of *rab7* (Figure 6A). Downregulation of *rab7* reflects a decrease in lipophagy [33]. Therefore, the inhibition of *rab7* reduced the degradation of LDs by autophagy (Figure 6B). Most Zn-induced upregulated autophagy-related genes, including atg1, atg4, lc3b, beclin1, and tfeb, were unaffected by CID. The autophagy genes detected are mainly involved in membrane and autophagosome formation [5,52]. *rab7* mainly promotes the fusion of the lysosome and autophagosome coated with lipid droplets [10]. Although these two parts together constitute lipophagy, they are relatively separate processes. Therefore, inhibition of *rab7* may not affect the formation of autophagosomes, implying that Zn-induced autophagy may also degrade other substances besides lipids. Zn has been reported to have the potential to protect against lipid overaccumulation and preserve lipid homeostasis [14,15,17,53,54]. In this study, 60 μM Zn downregulated lipogenesis-related genes and upregulated the mRNA levels of lipolysis-related genes (Figure 6C). However, the *rab7* inhibitor CID only mitigated the Zn-induced increase of *cpt1α* and acadm expression and had no effect on lipid synthesis genes. *Cpt1α* and *acadm* are the key genes of FA β-oxidation [14]. The results suggested that *rab7* inhibition may reduce the oxidative breakdown of FAs. Lipids can promote β-oxidation via the provision of FFA from LD degradation [5,27]. Given that *rab7* is essential for LD and lysosomal fusion, the inhibition of cpt1α and acadm may result from the inability of LDs to degrade into FAs through autophagy after *rab7* inhibition, leading to insufficient raw materials for β-oxidation. Taken together, the lipid-lowering effect of Zn can be partly attributed to *rab7*-dependent lipophagy and its promotion of FA β-oxidation in yellow catfish hepatocytes.

In summary, we delineated the *rab7* promoter region in yellow catfish and examined its interaction with FOXO3. We also studied the crucial role of FOXO3/*rab7* in Zn-induced lipophagy and the reduction of lipid deposition. This study reveals novel insights into FOXO3/rab7 role in lipophagy regulation, enhancing the understanding of lipid metabolism control. Meanwhile, this study indicates that in the culture of yellow catfish, lipid overaccumulation can be reduced by activating lipophagy and FA β-oxidation through the addition of appropriate Zn to the diet and promoting the healthy culture of yellow catfish.

## Data Availability

All data are contained within the article.

## Supplementary Materials

The following supporting information can be downloaded at: https://www.mdpi.com/article/10.3390/genes15030334/s1, Table S1: Primers used for Rab7 promoter cloning; Table S2: Primers used for 5′-deletion plasmids construction; Table S3: The reference binding site sequences; Table S4: Primers used for site-mutation analysis; Table S5: Primers used for Q-PCR analysis.
