# Supplementary material for: FOXO3/Rab7-Mediated Lipophagy and Its Role in Zn-Induced Lipid Metabolism in Yellow Catfish (Pelteobagrus fulvidraco)
Source: Genes (Basel). 2024 Mar 4;15(3):334. doi: 10.3390/genes15030334 (PMC10969980; doi:10.3390/genes15030334)
Supplement: Supplementary file 1 [file genes-15-00334-s001.zip › Supplementary Material .pdf]

Table S1. Primers used for Rab7 promoter cloning

Gene	Forward primer (5'-3')	Reverse primer (5'-3')	Step
Rab7	ACGATGGACTCCAGAGCGG CCGCVVNVNNNCCAA	GATCTACTGTATGAACT ATAACTC	hiTAIL -PCR1
	ACGATGGACTCCAGAG	ACAGTTTAACTGCCAGA AGTATCTGCT	hiTAIL -PCR2
	ACGATGGACTCCAGAG	CTTCAAGAGCAGCTTAC TTTCACAG	hiTAIL -PCR3

Table S2. Primers used for 5'-deletion plasmids construction

Gene	Primers	Forward primer (5'-3')	Reverse primer (5'-3')
Rab7	pGl3- -382/+67	ctatcgataggtaccgagctcTTAAA GAACTTTTTAACATATCA AC	cagtaccggaatgccaagcttTAACTC CCGTCTCTCACTTCCTGTTC
	pGl3- -795/+67	ctatcgataggtaccgagctcACCCT GTCACCCTAACCCTGTAA CC	cagtaccggaatgccaagcttTAACTC CCGTCTCTCACTTCCTGTTC
	pGl3- -1206/+67	ctatcgataggtaccgagctcCTAAC CCTGTAACCCTGTAACCC TG	cagtaccggaatgccaagcttTAACTC CCGTCTCTCACTTCCTGTTC
	pGl3- -1600/+67	ctatcgataggtaccgagctcAACCT CTCTAGGGGGAACCCCCT AA	cagtaccggaatgccaagcttTAACTC CCGTCTCTCACTTCCTGTTC

Table S3. The reference binding site sequences

Name	Binding site sequences
FOXO3	GTAAACA
FXR	AGGTCA
HNF4α	CAAAGTCCA
KLF4	AAAGGAAGG
PPARα/RXR	GGNAAAGGT
SREBP2	TCACGTCA
STAT3	TTCTNGGAA
TATA-box	TATAAA
TFEB	CACGTGAC
NRF2	TGACTCGCA

Table S4. Primers used for site-mutation analysis

Gene	Primers	Forward primer (5'-3')	Reverse primer (5'-3')
Rab7	Mut-FOXO3-1	CTTAcaccgatggaatAAAACAAATACTTACTTATTTATATTTCAATTGC	TTattccatcggtgTAAGAATAGTGTTGTGTGTTCCATGTATG
	Mut-FOXO3-2	GTTATTtcagcgtcatgaTATTTCAAAAATCCTTGTGATATATTCAGT	tcatgacgctgaAATAACAAAACGAATAACAAAATAAACAA
	Mut-FOXO3-3	TTTgatcctgacgaaGTTATTCGTTTTGTTATTTATTATTTACTATATTTC	AACttcgtcaggatcAAAATAACAAAAATAAATAAATAACAAAACAA

Table S5. Primers used for Q-PCR analysis

Gene	Forward primer (5'-3')	Reverse primer (5'-3')
6pgd	CTGCTGCTGGACTCCTTCTT	GTTGTGTCTGTAACCGTCGTAA
acadm	GCAGAAGGAGTTCCAGGAGGTGTC	CAGCAATAATGACCGGCATTTGTC
accα	ACTTCTGCTGTGGTTGTCCTAT	GCATCCATCGTGGGTCATA
atg1	ATCTGGTGAAGGTGCTGAAGT	CTCTGACTGTGGCAGGTTGT
atg3	GGAAGGCTGAGGCAAGCGGAGA	TGGGTGGTTTTCAATGGTGACTGTTTT
atg4	ATGGAGGCAGTTTTAGCCAAGTAT	TGTATGTAAACCACAGCCGTGAA
atg5	CAGAACCGTTTTATCTTCTCCTACCG	CGTCTACATCTTCAGCTTTCACGACTT
atgl	ATCTGGTGAAGGTGCTGAAGT	CTCTGACTGTGGCAGGTTGT
β-actin	GTGCGTGACATCAAGGAGAAG	CGAGGAAGGATGGCTGGAA
b2m	GCTGATCTGCCATGTGAGTG	TGTCTGACACTGCAGCTGTA
beclin1	CTCAACTGGACCGCCTGAAGAA	CACTCCACAGGAACGCTGGGTA
cpt1α	CGCTCCTGCTCCAATGAGA	GAGACCACATAGAGGCAGAAGA
elfa	GTCTGGAGATGCTGCCATTG	AGCCTTCTTCTCAACGCTCT
fas	CATCATCACTGGAGGTCTTGGA	TACGAATGCCTGATCTGGAAGT
foxo3	CCCGTGTTTACCTTCAGCTG	TCAATGGGTCAGACTGGGTC
g6pd	GAGAAGCCTGCCTCAACCA	GGATCGTCCAAGTAGCCAAGT
gapdh	AAAGTCATCCCCGAGCTCAA	CTTCAGACGCAGCCTTCATC
hprt	ATGCTTCTGACCTGGAACGT	TTGCGGTTCAGTGCTTTGAT

hsl	ACCATTGCTCCACCGTCTG	CGTCTCACTATCCTGTCCTTCA
lamp	CAGCAACAGCAGTAGTGGGA	AGCTCAGTGTAAGGTTGGCC
lc3b	CCTGACCACGTCAACATGAGCGAACT	GGAAATGGCGGCAGACACGGAGA
rab7	AAGCTTTCCAGACCATCGCA	AGAGAGGGTGGAGGGTGATC
rpl7	GCGCCAGATCTTCAATGGAG	CTCATTCTGCCATGACCACG
tbp	AGTCCCATGATGCCCTATGG	GCAACAGCTTGGGAATGGAA
tfeb	ACCAGCGACCTCCTCCTAAT	AGCTCAAATCTCCCAGGCAC
tuba	CACTTCCCTCTTGCCACCTA	ACGGTACAGGAGACAACAGG
